# B-type natriuretic peptide attenuates TLR-induced cytokine and chemokine secretion in monocyte-derived Langerhans cells

**DOI:** 10.3389/fimmu.2026.1791284

**Published:** 2026-03-19

**Authors:** Dorottya Horváth, Zsófia Pénzes, Petra Molnár, Szabolcs Muzsai, Magdolna Szántó, Andrea Szegedi, Anikó Kapitány, Attila Bácsi, Attila Gábor Szöllősi

**Affiliations:** 1 Department of Immunology, Faculty of Medicine, University of Debrecen, Debrecen, Hungary; 2 Doctoral School of Molecular Medicine, University of Debrecen, Debrecen, Hungary; 3 Gyula Petrányi Doctoral School of Clinical Immunology and Allergology, University of Debrecen, Debrecen, Hungary; 4 Department of Medical Chemistry, Faculty of Medicine, University of Debrecen, Debrecen, Hungary; 5 MTA-DE Lendület “Momentum” Skin Immunometabolism Research Group, Debrecen, Hungary; 6 HUN-REN-DE Allergology Research Group, Debrecen, Hungary; 7 Department of Dermatology, MTA Centre of Excellence, Faculty of Medicine, University of Debrecen, Debrecen, Hungary

**Keywords:** B-type natriuretic peptide, chemokines, migration, monocyte-derived Langerhans cell, neuroimmune interaction, neuropeptide, skin immunology

## Abstract

**Introduction:**

B-type natriuretic peptide (BNP) is a well-known cardiac hormone and biomarker of heart failure, but emerging evidence suggests that it also possesses immunomodulatory properties, including a role in inflammatory skin conditions like atopic dermatitis (AD). Langerhans cells (LCs), specialized epidermal antigen-presenting cells, orchestrate cutaneous immunity and are targets of neuropeptides. In the present study, we investigated how BNP treatment during differentiation affects the activation, cytokine profile, and interaction of immune cells with moLCs subsequently activated via Toll-like receptors (TLRs).

**Methods:**

MoLCs were differentiated in the presence or absence of BNP, followed by 24-hour activation with the TLR7/8 agonist CL075 and/or the TLR3 agonist polyinosinic:polycytidylic acid (poly(I:C)). Cell surface markers of moLCs were assessed using flow cytometry. ELISA was used to analyze the production of cytokines. The T cell proliferation-inducing ability of moLCs was detected through T cell coculture. Transwell migration experiments were conducted to elucidate the migratory capacity of moLCs, as well as the migration of other lymphocytes toward moLCs.

**Results:**

BNP treatment during the differentiation of moLCs did not alter the expression of activation markers; however, it significantly counteracted the robust increase in both pro- and anti-inflammatory cytokine production induced by TLR activation. Combined TLR activation significantly increased T cell proliferation capacity, and this effect was significantly diminished in moLCs differentiated in the presence of BNP. Functionally, BNP pre-treated moLCs exhibited significantly enhanced chemotaxis towards the lymph node chemokines, supporting the previously observed migratory phenotype. Transcriptomic analysis further supported this finding, demonstrating that BNP pre-treatment attenuated the inflammatory gene signature induced by TLR agonism. Furthermore, the supernatant from TLR-activated, BNP-treated moLCs showed a marked reduction in the ability to induce the migration of peripheral blood CD56^+^ Natural Killer (NK) cells.

**Discussion:**

We found that BNP primes moLCs toward a migratory phenotype and, upon subsequent TLR activation, exerts a potent inhibitory effect on their cytokine and chemokine production, thereby limiting their capacity to drive T cell proliferation and NK cell migration. This dual effect suggests that BNP may play a context-dependent role in skin immunity, potentially restraining inflammation while promoting LC transit to the draining lymph nodes.

## 1 Introduction

B-type natriuretic peptide (BNP) was first described as a mediator secreted by the ventricular cardiomyocytes. The main function of BNP is to regulate blood pressure and fluid volume by causing vasodilation. BNP is a 32-amino acid cyclic peptide that binds to natriuretic peptide receptor 1 (NPR1) and the silent receptor NPR3 (1). NPR1 is expressed in multiple tissues such as the kidneys, lungs, and adrenal glands (2). Moreover, *in vitro* studies have demonstrated its expression in vascular endothelial cells and renal epithelial cells. It is also highly expressed in smooth muscle cells from the aortic tunica media (3, 4). The binding of NPR1 induces cyclic guanosine monophosphate (cGMP) synthesis. Rising cGMP levels promote cGMP-dependent phosphodiesterases, ion channels, and protein kinases (PKG-I and -II) (1). BNP binding to NPR1 results in intracellular cGMP accumulation and PKG-I activation, which prevents IL-1β release by inhibiting the assembly of the inflammasome (5).

BNP is well known as a biomarker during heart failure as its level is highly increased in the patients’ circulation (6). It counterbalances the chronic activation of the sympathetic nervous system, the renin-angiotensin-aldosterone (RAA) system, and the arginine-vasopressin system by causing natriuresis, diuresis, and vasodilation (7, 8). Accordingly, BNP concentration in the serum of patients can establish the severity of heart failure and support the diagnosis (8).

Besides its primary role in cardiovascular mechanisms, BNP has recently been shown to have immunomodulatory functions (4, 9–12). In a mixed lymphocyte reaction (MLR), the inhibitory effect of BNP on T cell proliferation was determined (13). In THP-1 macrophage cells, BNP increased reactive oxygen species (ROS), nitrite (NO_2_), prostaglandin E_2_ (PGE_2_), and leukotriene B_4_ (LTB4) production and increased the migratory capability of THP-1 macrophages in a wound healing assay.

Moreover, it has an important role in itch transmission in the central and peripheral nervous systems (14). During atopic dermatitis (AD), BNP is released from peripheral nerve endings in response to an IL-31 stimulus and contributes to itch formation and exacerbates the inflammatory environment by activating keratinocytes (15). In an AD mouse model induced by the vitamin D agonist MC903, BNP-KO mice exhibited significantly decreased scratching responses, and skin thickening compared to wild-type (WT) mice, which means BNP could play a role in AD-associated inflammation (16).

AD has a very complex pathophysiology mediated by T cells. During the acute phase of the disease, T helper 2 cells (Th2 cells) predominate and secrete IL-4 and IL-13. These cytokines and their receptors are targets for treatment of AD, as shown by the efficacy of, for example, dupilumab. This biologic targets the common receptor subunit of both cytokines (IL-4Rα), and is one of the most effective therapies in moderate-to-severe AD to date (17, 18). In AD patients, keratinocytes release high levels of thymic stromal lymphopoietin (TSLP) in lesions, a cytokine that promotes Langerhans cells (LCs) to produce CCL17 and CCL22 chemokines. These chemokines recruit T cells and polarize them to Th2 phenotype (18–21).

LCs are a type of antigen-presenting cell in the epidermal layer of the skin, which are likely a target of neuropeptides originating from the epidermis (22). LCs not only have an immunostimulatory role but are also crucial in immunoregulation (23). They originate from embryonic macrophage precursors and seed the skin before birth (24, 25). Another property that makes them similar to macrophages is that they sustain themselves locally with slow proliferation activity without mobilizing cells from the bone marrow. Inflammatory conditions increase the migration of LCs, causing cell depletion, and a subset of monocytes helps to restore them with differentiation to steady-state LCs (26–28). They also have characteristics of dendritic cells. Using long dendrites, they effectively monitor the environment between tight junctions (29, 30). During steady state or inflammation, they can migrate to the lymph nodes with the aim of antigen presentation to T cells. This requires the loss of their adhesion to the surrounding keratinocytes, including the downregulation of E-cadherin (28, 31).

Our previous *in vitro* experiments demonstrate that BNP influences the differentiation and promotes the development of a migratory LC phenotype in monocyte-derived LCs (moLCs), as well as upregulating genes related to cell motility (32). However, the effect of BNP-conditioned LCs on immune responses remains unclear. Our goal in the current study is to investigate the effect of BNP on the migration and interaction of Toll-like receptor (TLR) activated LCs with other immune cells.

## 2 Results

### 2.1 Combination of TLR agonists effectively increased activation markers (CD83, CD86 and HLA-DQ) expression in moLCs

**Figure 1:**
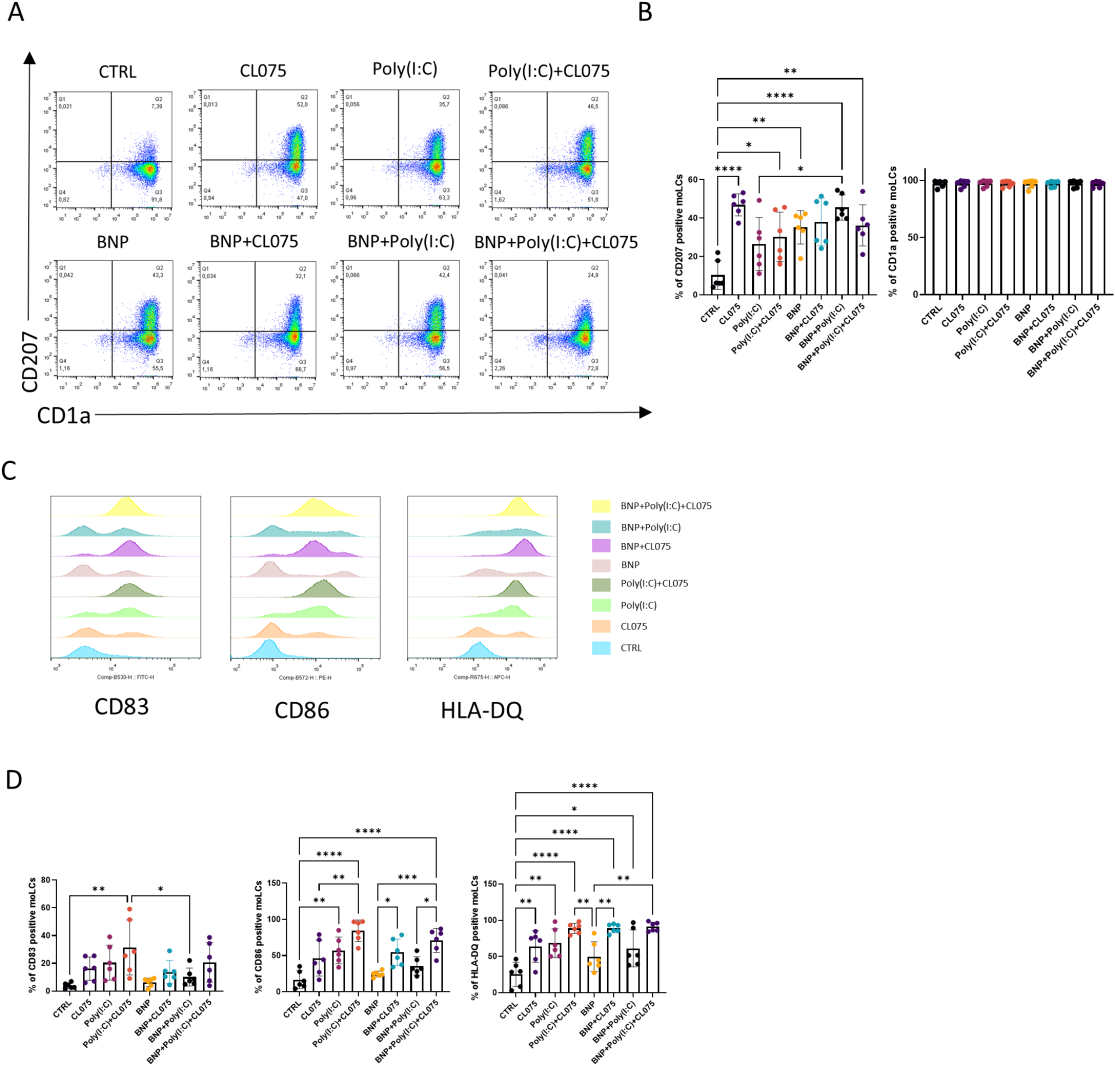
The effects of BNP on activated moLCs differentiation and activation markers. **(A)** Representative density plot of the main markers of moLCs, CD1a and CD207 expression measured by flow cytometry. Monocytes were cultured in the presence of GM-CSF, TNF-α, and TGF-β for 5 days supplemented with IL-4 for the first 48 hrs to differentiate moLCs, and treated with 10 nM BNP throughout the entire differentiation process. On day 4 moLCs were activated with CL075 and poly(I:C) and a combination of both for 24 hrs. **(B)** A percentage of positive CD207 and CD1a moLCs. N = 6 **(C)** Histograms of moLCs activation markers, CD83, CD86, and HLA-DQ. **(D)** A percentage of positive CD83, CD86 and HLA-DQ moLCs. Symbols with different colors represent individual donors. Data are presented as individual values with mean ± SEM. Statistical significance was determined by One-way ANOVA followed by Tukey’s *post-hoc* test. Homogeneity of variances was confirmed by the Brown-Forsythe test. n=6 biological replicates per group. *P<0.05, **P<0.01, *** P<0.001, **** P< 0.0001. BNP, B-type natriuretic peptide; CL075, TLR7/8 agonist, thiazoquinoline compound; CTRL, Control; Poly(I:C), TLR3 agonist, polyinosinic:polycytidylic acid.

To measure the effects of BNP on moLC differentiation and activation, we used flow cytometry for the analysis of the two main characteristic differentiation markers, CD207 and CD1a (**Figures 1A, B**), as well as markers of activation CD83, CD86, and HLA-DQ (**Figure 1C**, gating strategy and representative dot plot are shown in **Supplementary Figure 1**). The expression of langerin (CD207) was increased after activation with CL075, which resulted in the highest proportion of CD207-positive cells. Using BNP during differentiation also resulted in a high level of CD207 positive cells (consistent with our previous findings (32)) and further increased the ratio of positive cells when combined with polyinosinic:polycytidylic acid (poly(I:C)) treatment (**Figure 1B**). Combined TLR activation resulted in CD207 expression levels comparable to those observed with individual TLR agonist treatments (**Figure 1B**). The percentage of CD1a positive cells was consistently high and remained unchanged either by the presence of BNP during the differentiation of the cells, or by the subsequent activation of moLCs with TLR agonists CL075 and poly(I:C); agonists of TLR7/8 and TLR3, respectively; **Figures 1A, B**).

Both TLR ligands could increase the percentage of moLCs expressing the activation markers CD83, CD86, and HLA-DQ, although when applied alone poly(I:C) was proved to be more effective (**Figures 1C, D**). MoLCs differentiated in the presence of BNP alone did not show any altered marker expression, but rather decreased the impact of poly(I:C) in a non-significant manner (**Figures 1C, D**). The greatest effects were achieved by the combination of TLR ligands (**Figures 1C, D**), which significantly increased all activation markers compared to the control sample (**Figure 1D**). BNP did not interfere with this activation-induced upregulation. These findings were further supported by analyses of mean fluorescent intensity (MFI) and ΔMFI values, which confirmed the significant effects of the combined TLR agonists (**Supplementary Figure 2**). A71915, a highly potent and competitive antagonist of NPR1 (33, 34) was used to test BNP achieves these effects through NPR1 on the cells’ differentiational and activation markers. BNP, in the presence of the NPR1 antagonist, failed to increase the ratio of CD207^+^ cells. The proportion of activation markers such as CD83, CD86 and HLA-DQ was also low, as seen in the control samples (**Supplementary Figure 3**).

### 2.2 MoLCs differentiated in the presence of BNP show an attenuated cytokine response to TLR agonist stimulation

**Figure 2:**
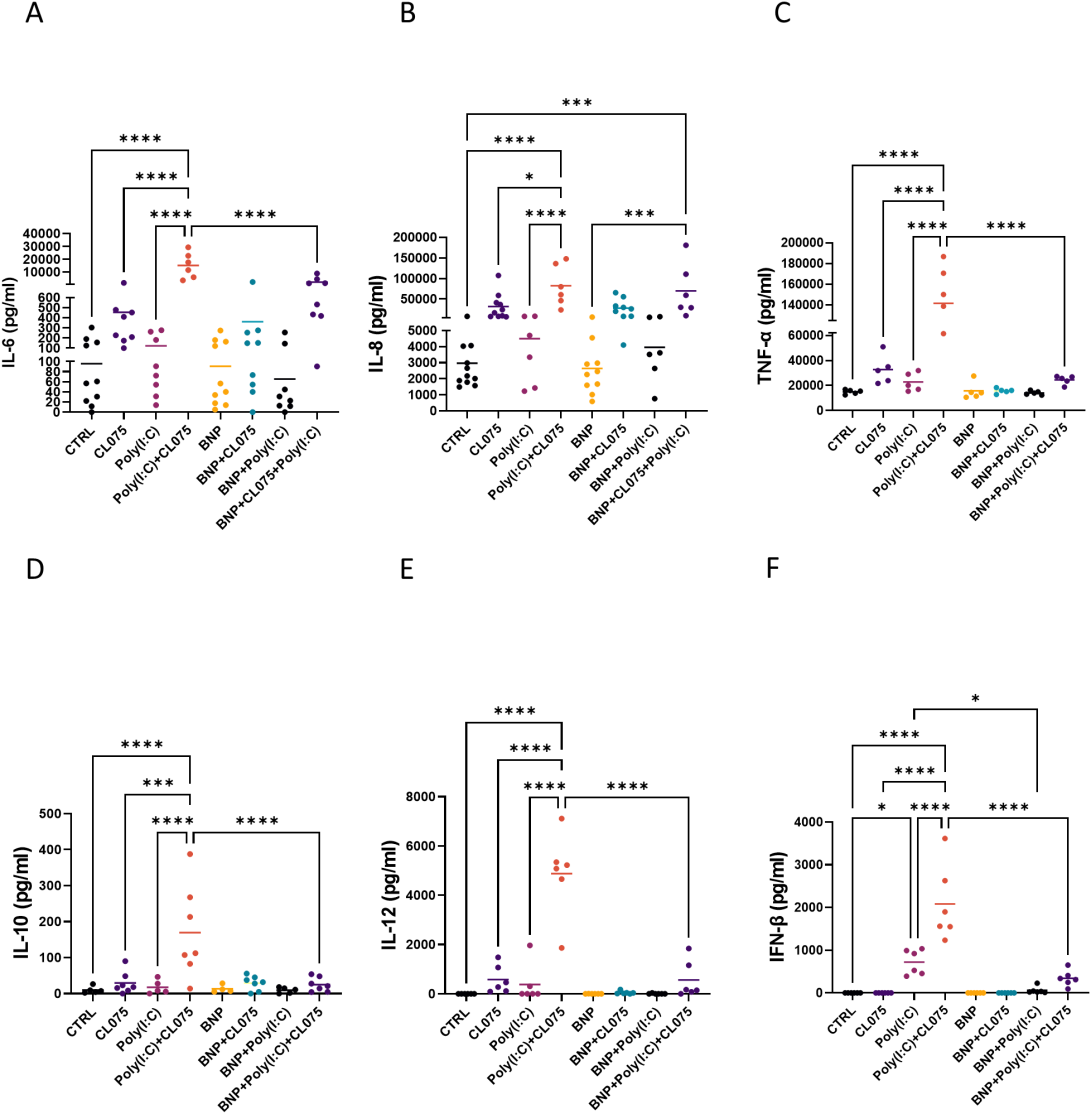
BNP inhibited the TLR activated moLCs IL-6, TNF-α, IL-12, IL-10 and IFN-β cytokine production. Monocytes were cultured in the presence of GM-CSF, TNF-α, and TGF-β for 5 days supplemented with IL-4 for the first 48 hrs to differentiate moLCs, and treated with 10 nM BNP throughout the entire differentiation process. On day 4 moLCs were activated with CL075 and poly(I:C) and a combination of both for 24 hrs. Supernatants were collected on day 5 from the cells. **(A–F)** Levels of different pro-inflammatory cytokines IL-6 and IL-8, TNF-α, cytokines during viral response IL-12 and IFN-β, and an anti-inflammatory IL-10, were measured by ELISA. Symbols with different colors represent individual donors, lines marks mean expression levels. Data is presented as Mean ± SEM. Two-way ANOVA followed by Tukey’s multiple comparison test. Normality of residuals was confirmed by QQ plot analysis. n=6–10 biological replicates per group *P<0.05, **P<0.01, *** P<0.001, **** P< 0.0001. BNP, B-type natriuretic peptide; CL075; TLR7/8 agonist, thiazoquinoline compound; CTRL, Control; moLC, monocyte-derived Langerhans cells; Poly(I:C), TLR3 agonist, polyinosinic:polycytidylic acid.

We determined the effect of the NPR1 agonist applied during differentiation on cytokine production by moLCs, using ELISA assays. We checked the capacity of moLCs to produce pro-inflammatory cytokines i.e., IL-6, IL-8, and TNF-α (**Figures 2A–C**). The TLR agonist poly(I:C) and CL075 treatment alone increased the production of all three cytokines compared to the vehicle, while combined stimulation resulted in the strongest induction. The presence of BNP during the differentiation of the cells did not affect these cytokine concentrations alone, but could counteract some of the effects of TLR agonists. Namely, BNP significantly reduced the levels of IL-6 and TNF-α when combined activators were added to the cells (**Figures 2A, C**). In contrast BNP was unable to modify the IL-8 concentration (**Figure 2B**), which is produced in large amounts by the activation. After determining the diminishing effects of BNP on activated moLCs’ pro-inflammatory cytokine production, we decided to investigate its influence on the production of anti-inflammatory cytokine IL-10 (**Figure 2D**). IL-10 levels increased after treatment with TLR agonists, and BNP also had an inhibitory effect on this (**Figure 2D**). Cytokines generated upon viral infection, IL-12 and IFN-β, were strongly induced by poly(I:C) and CL075, and cells differentiated in the presence of BNP showed a significant decrease in the production of these cytokines as well (**Figures 2E, F**). Overall, BNP has a marked inhibitory effect on the cytokine production induced by TLR activation in moLCs.

### 2.3 BNP inhibits the T cell proliferation-inducing capacity of activated moLCs independently of IDO1

**Figure 3:**
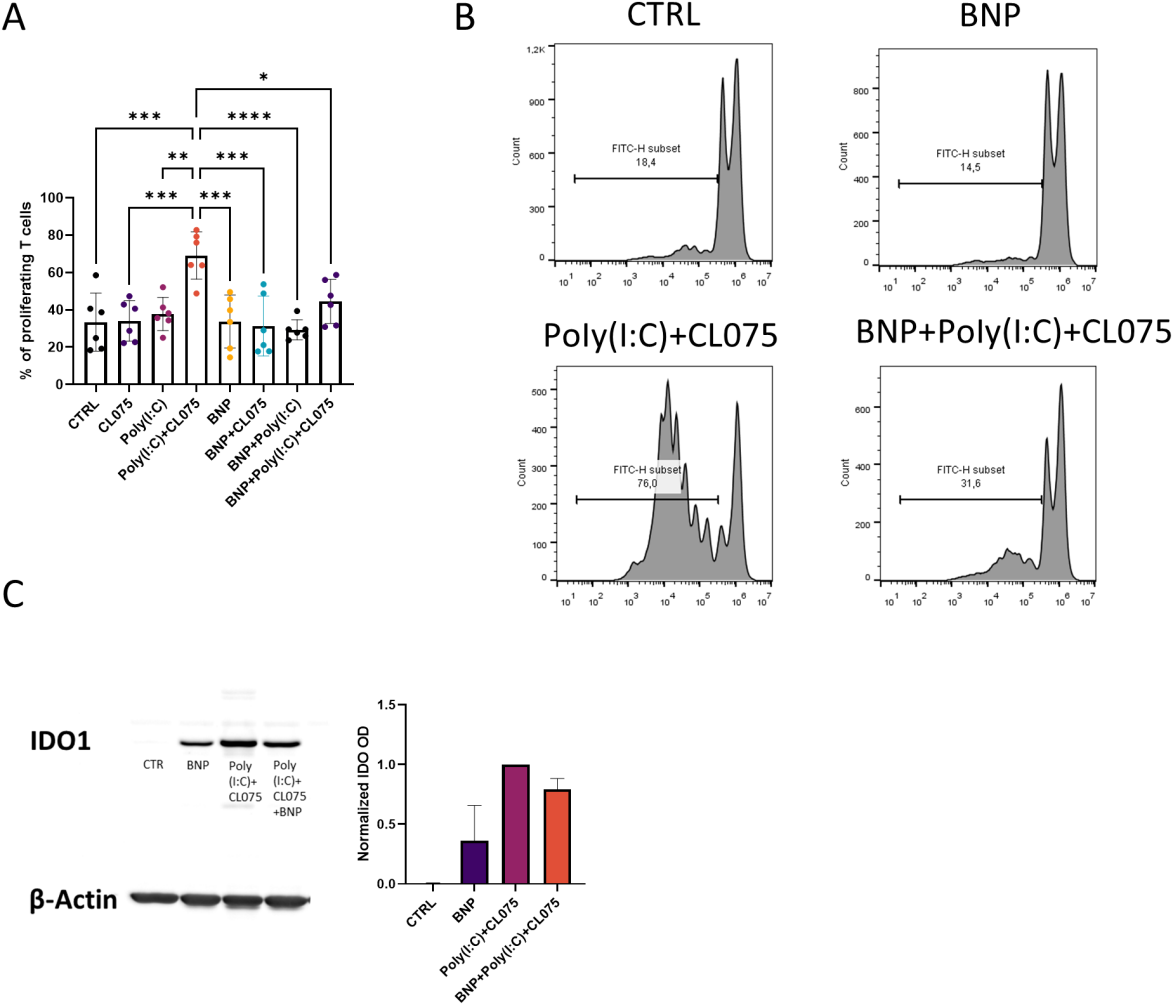
BNP reduced the T cell proliferation induction ability in Poly(I:C) and CL075 activated moLCs. **(A)** Proliferating T cells were measured with flow cytometry by the CFSE intensity at day 5 of coculture. Monocytes were cultured in the presence of GM-CSF, TNF-α, and TGF-β for 5 days, supplemented with IL-4 for the first 48 hrs to differentiate moLCs, and treated with 10 nM BNP throughout the differentiation process. The moLCs were activated for 24 hrs with CL075, and poly(I:C), and a combination of both. Naїve CD4^+^ T cells were isolated with CD4^+^ magnetic beads and made a coculture with fully differentiated moLCs for 5 days. n=6 **(B)** Representative histograms of CFSE intensity from a coculture of T cells and moLCs. **(C)** Representative Western blot of IDO1 expression of moLCs. Cells were treated with 10 nM BNP and activated for 24 hrs in the presence of CL075 and poly(I:C) at day 4. Symbols with different colors represent individual donors. Data are presented as individual values with mean ± SEM. Statistical significance was determined by One-way ANOVA followed by Tukey’s *post-hoc* test. Homogeneity of variances was confirmed by the Brown-Forsythe test. n=2 biological replicates per group *P<0.05, **P<0.01, *** P<0.001, **** P< 0.0001. BNP, B-type natriuretic peptide; CFSE, Carboxyfluorescein succinimidyl ester; CL075; TLR7/8 agonist, thiazoquinoline compound; CTRL, Control; IDO1, Indoleamine 2,3-dioxygenase; Poly(I:C), TLR3 agonist, polyinosinic:polycytidylic acid.

LCs have a crucial role in presenting antigen to naïve T cells in draining lymph nodes and, thereby controlling the maintenance of skin immunity. Therefore, we investigated the effect of BNP on the coculture of activated moLCs and T cells. The capability of moLCs to induce T cell proliferation did not change after differentiating the cells in the presence of BNP or by TLR agonists applied alone compared to the control sample. Combined poly(I:C) and CL075 increased this ability and significantly changed the percentage of proliferating T cells. However, this ability was significantly decreased by BNP (**Figures 3A, B**). Our previous RNA-Seq results (32) showed that BNP increased indoleamine 2,3-dioxygenase 1 (IDO1) expression in moLCs. To assess whether the decrease in T cell proliferation could be due to IDO1 induction, we examined IDO1 protein levels with Western blot. Although BNP increased IDO1 expression, a comparable upregulation was also observed following poly(I:C) and CL075 stimulation (**Figure 3C**). These findings indicate that the BNP-mediated suppression of T cell proliferation occurs independently of IDO1 protein expression levels (**Figure 3C**).

### 2.4 BNP attenuates the inflammatory gene signature induced by combined TLR agonism

Our previous transcriptomic analysis sought to define the impact of BNP on the differentiation of moLCs. We found that while BNP treatment during differentiation did not fundamentally alter the moLC subtype identity, it did induce a significant transcriptional shift towards a migratory phenotype. (32) This was evidenced by the upregulation of key migratory genes such as CCR7 and LAMP3, as well as genes implicated in T cell regulation like IDO1. Consistently, gene set enrichment analysis (GSEA) confirmed that the most significantly enriched pathways in BNP-treated moLCs were those associated with cell migration, motility, and locomotion.

**Figure 4:**
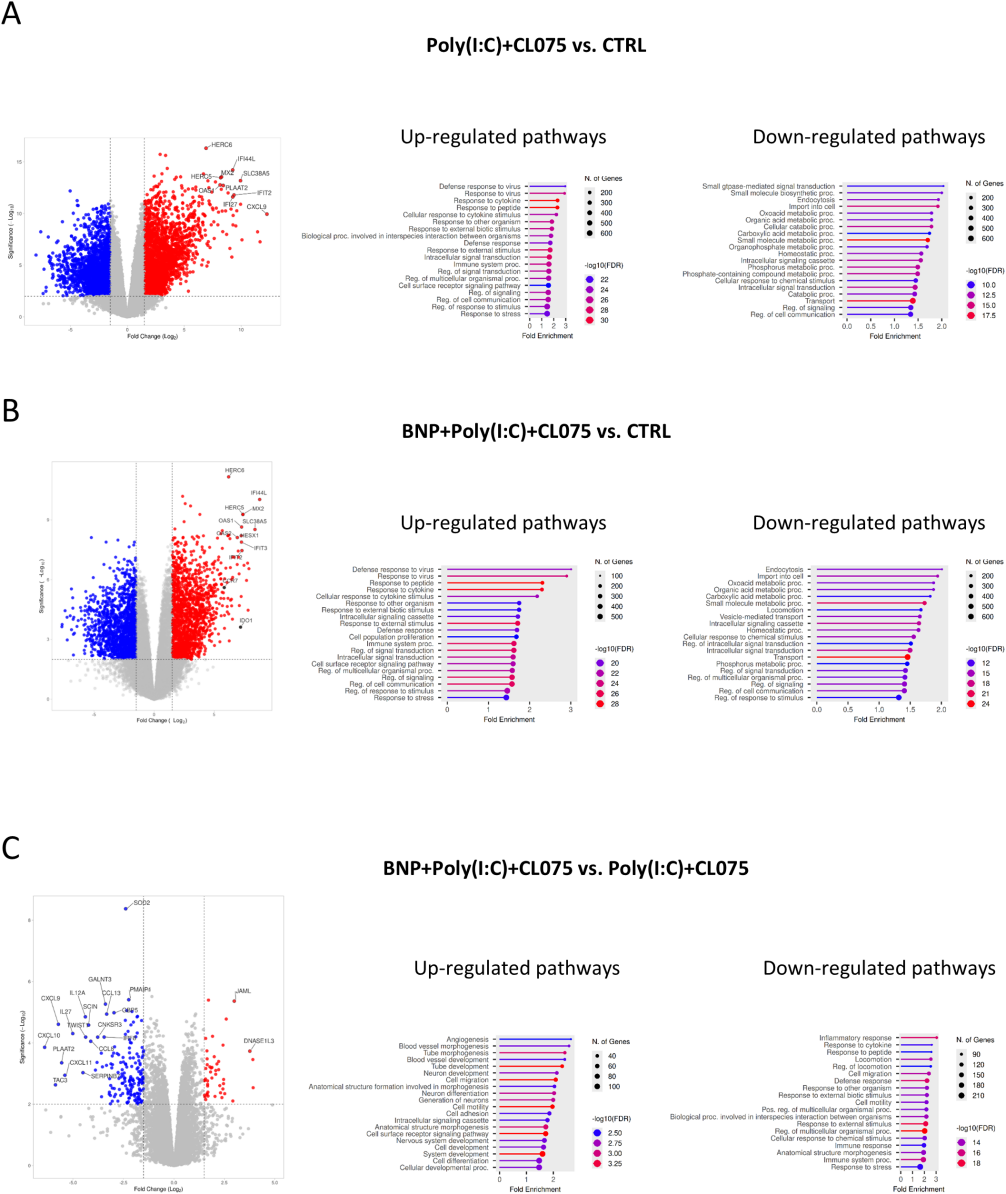
RNA-Seq analysis showed an upregulation of migratory genes and the downregulation of inflammatory cytokines and chemokines in activated moLCs. Monocytes were cultured in the presence of GM-CSF, TNF-α, and TGF-β for 5 days supplemented with IL-4 for the first 48 hrs to differentiate moLCs, and treated with 10 nM BNP throughout the differentiation process. The moLCs were activated for 24 hrs with CL075, and poly(I:C), and a combination of both. [(**A–C)**, left] Volcano plot analysis of poly(I:C) and CL075 and BNP treated moLCs compared to vehicle-treated controls or each other. The top 15 most significantly changed gene symbols are highlighted. [**(A–C**, middle, right] GO annotation of the biological process of up- and down-regulated genes between the groups marked. The size of the circle corresponds to the number of induced genes, while the color of the line corresponds to the -log10 of the False Discovery Rate (FDR). **(A)** Poly(I:C) and CL075 vs. CTRL **(B)** BNP, Poly(I:C) and CL075 vs. CTRL **(C)** BNP, Poly(I:C) and CL075 vs. Poly(I:C) and CL075. BNP, B-type natriuretic peptide; CL075; TLR7/8 agonist, thiazoquinoline compound; CTRL, Control; GO, gene ontology; Poly(I:C), TLR3 agonist, polyinosinic:polycytidylic acid.

Building on these findings, we next investigated the effect of BNP treatment during differentiation on the subsequent activation state of the cells. As expected, activation of control moLCs with TLR7 and TLR8 agonists led to a robust upregulation of genes involved in the antiviral response, including HERC6, IFI44L, MX2, IFIT2, CXCL9, IFI27, and OAS1 (**Figures 4A**, left). While moLCs differentiated in the presence of BNP showed a nearly identical pattern of gene induction upon activation, the magnitude of this upregulation was consistently less pronounced (**Figures 4B**, left). Notably, the expression of CCR7 and IDO1 was upregulated in both activated populations compared to their respective unstimulated controls (**Figures 4A, B**, left).

GSEA of the activated moLCs versus their unstimulated controls supports these observations. In both the control and BNP-differentiated groups, the most highly upregulated pathways upon activation were related to “Defense response to virus,” “Response to cytokine,” and “Immune system processes” (**Figure 4A**, middle). Conversely, downregulated pathways in both groups were primarily associated with metabolic activity, such as “Oxoacid metabolic process” and “Carboxylic acid metabolic process” (**Figure 4A**, right). However, “Locomotion” also appeared as a significantly downregulated pathway in the activated BNP group (**Figure 4B**, right), an effect not observed in the activated control cells (**Figure 4A**, right).

Finally, a direct comparison between the two activated populations (control vs. BNP-differentiated) revealed that more genes were downregulated than upregulated (732 vs. 398) in the BNP-treated group. The downregulated gene set included several notable inflammatory cytokines and chemokines, such as CXCL9, CXCL10, IL12A, CCL8, CXCL11, and CCL13 (**Figure 4C**, left). GSEA of this direct comparison highlighted that while pathways related to migration and motility were present in both the up- and downregulated sets (**Figure 4C**, middle and right), the most significantly downregulated pathway in the BNP-treated activated cells was “Inflammatory response” (**Figure 4C**, right).

### 2.5 NPR1 agonist enhances moLC migration toward CCL21 while limiting chemokine production of activated cells

**Figure 5:**
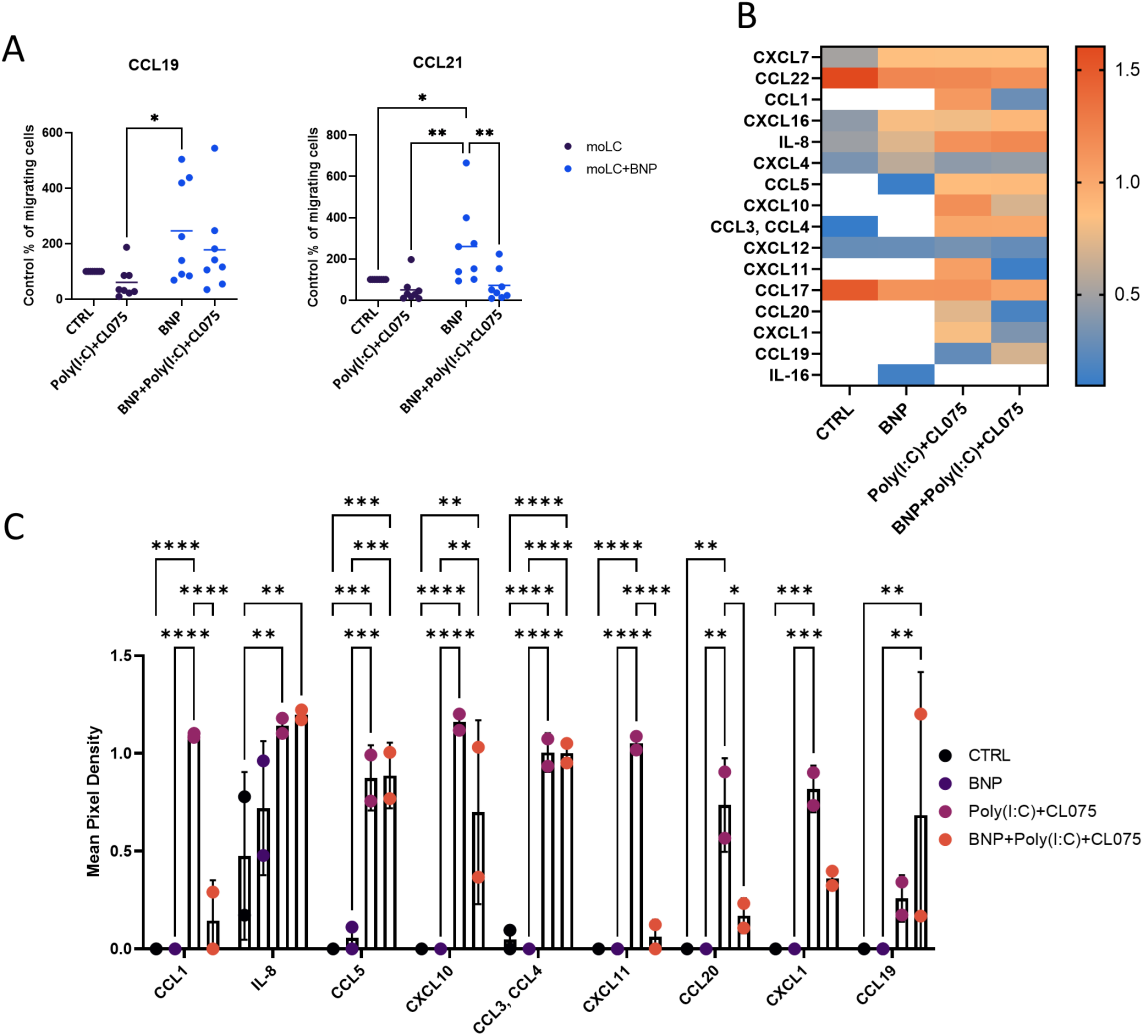
BNP treatment increased moLCs migration toward CCL21 and reduced chemokine production in activated moLCs. Monocytes were cultured in the presence of GMCSF, TNF-α, and TGF-β for 5 days supplemented with IL-4 for the first 48 hrs to differentiate moLCs, and treated with 10 nM BNP throughout the entire differentiation process. On day 4, moLCs were activated with CL075 and poly(I:C) and a combination of both for 24 hrs. **(A)** In Transwell migration assay, 1*10^6^ cells were applied in the upper well of the Transwell plate, and in the bottom of the plate, it contains RPMI supplemented with CCL19 and CCL21. Migration of the cells was measured with flow cytometry. Data are presented as individual values with mean ± SEM. Statistical significance was determined by Two-way ANOVA followed by Tukey’s multiple comparison test. Normality of residuals was confirmed by QQ plot analysis. n=8 biological replicates per group **(B)** A chemokine array was performed using supernatants from BNP treated and TLR activated cells. Symbols with different colors represent individual donors. Data is presented as Mean ± SEM. One-way ANOVA with Tukey’s multiple comparison test was used for statistical analysis. n=2 biological replicates per group *P<0.05, **P<0.01, *** P<0.001, **** P< 0.0001. BNP, B-type natriuretic peptide; CL075; TLR7/8 agonist, thiazoquinoline compound; CTRL, Control; moLC, monocyte-derived Langerhans cells; Poly(I:C), TLR3 agonist, polyinosinic:polycytidylic acid.

Since our RNA-Seq results showed that BNP impacts the motility of the cells, and because both BNP and TLR agonists upregulated CCR7 expression we next assessed the receptor’s functionality, using a Transwell migration platform. Our results showed that more cells migrated toward CCL19 and CCL21 when the cells were differentiated in the presence of BNP compared to control samples (**Figure 5A**). Poly(I:C) and CL075 did not alter the migration capability of moLCs; however they reduced the migration of the BNP samples.

### 2.6 BNP reduces chemokine production by TLR-activated moLCs

Based on the RNA-Seq results, we further investigated how BNP affects the chemokine production of the moLCs in the activated state of the cells. A chemokine array was performed from the moLCs’ supernatant to determine the most important secreted chemokines (**Figures 5B, C**). BNP alone did not show a significant effect on the chemokine production of moLCs compared to the control sample, while TLR activated cells produced significantly more chemokines, including CCL1, IL-8, CCL5, CXCL10, CCL3, CCL4, CXCL11, CCL20, CXCL1, and CCL19 (**Figures 5B, C**). This production was less pronounced when moLCs were treated with BNP during the differentiation process. In these cells the secretion of multiple chemokines was significantly reduced, including CCL1, CXCL11, CXCL1, and CCL20 (**Figures 5B, C**).

### 2.7 BNP reduces the ability of activated moLCs to induce CD56^+^ NK cells migration

**Figure 6:**
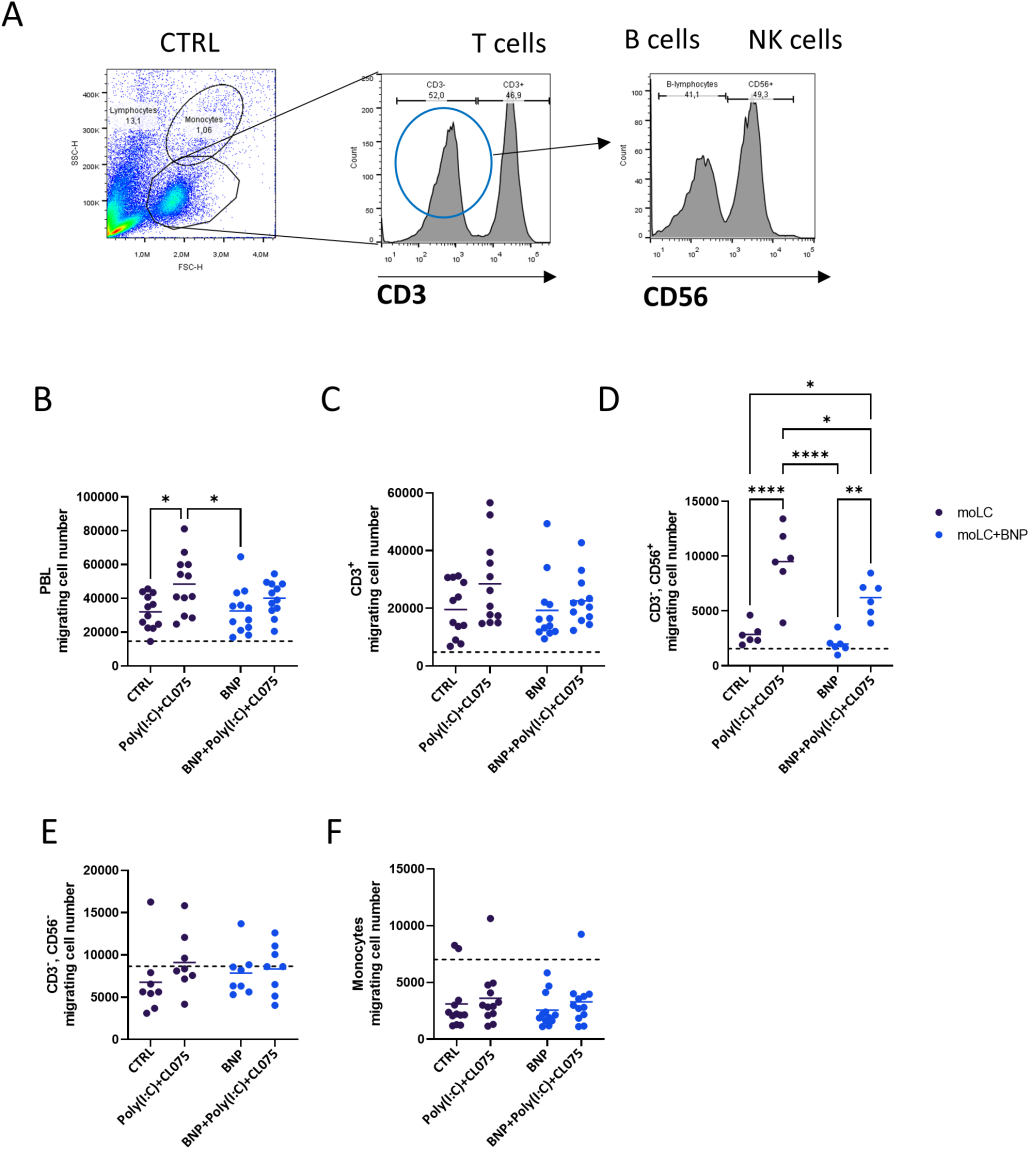
BNP treated moLCs supernatant inhibited NK cell migration. Monocytes were cultured in the presence of GM-CSF, TNF-α, and TGF-β for 5 days supplemented with IL-4 for the first 48 hrs to differentiate moLCs, and treated with 10 nM BNP throughout the entire differentiation process. On day 4 moLCs were activated with CL075 and poly(I:C) and a combination of both for 24 hrs. Transwell plates were used to perform the migration experiments, and cell numbers were determined by flow cytometry. **(A)** A flow cytometry gating strategy was used to distinguish between the PBL and monocyte populations based on the FSC-H and SSC-H. In the histograms, CD3^+^ cells represent the T cell population. CD3^-^ cells selected to determine the CD56^+^ population, which represented the NK cells. **(B–F)** Flow cytometry was used to measure the number of migrating cells in the lower wells of the Transwell plate. **(B)** PBL, **(C)** CD3^+^, **(D)** CD3^-^, CD56^+^, **(E)** CD3^-^, CD56^-^, **(F)** monocytes. Symbols represent individual donors. Data are presented as individual values with mean ± SEM. Statistical significance was determined by Two-way ANOVA followed by Tukey’s multiple comparison test. Normality of residuals was confirmed by QQ plot analysis. n=10–12 biological replicates per group *P<0.05, **P<0.01, **** P< 0.0001. BNP, B-type natriuretic peptide; CL075; TLR7/8 agonist, thiazoquinoline compound; CTRL, Control; moLC, monocyte-derived Langerhans cells; NK cells, Natural Killer cells; Poly(I:C), TLR3 agonist, polyinosinic:polycytidylic acid.

To test the effect of these chemokines on immune cells originating from the moLCs supernatant, we performed Transwell experiments using human peripheral blood lymphocytes (PBL). PBL populations were first gated based on their forward and side scatter. Using a CD3^+^ antibody, T cells were separated from other types of lymphocytes, while a CD56^+^ antibody was used to distinguish NK cells from B cells (**Figure 6A**). Supporting the chemokine array results, the PBL population migrated more toward the poly(I:C) and CL075 activated moLCs supernatant (**Figure 6B**). A similar pattern occurred with CD3^+^ population, which represents the T cells, but showed no statistically significant difference (**Figure 6C**). Cells differentiated in the presence of BNP showed a general tendency to elicit less migration as seen by the overall downward trend in the lines connecting the samples from the same donors (**Figures 6B, C**), but this effect was not statistically significant. The CD3^-^ population is comprised of NK and B cells, which we differentiated based on their expression of CD56 (**Figures 6D, E**). CD3^-^ CD56^+^ NK cells exhibited a markedly enhanced migratory potential towards the supernatant of activated moLCs. Importantly, this NK cell migration was significantly attenuated when supernatants were derived from moLCs differentiated in the presence of BNP and subsequently activated with TLR agonists (**Figure 6D**). In contrast, no significant difference was observed compared to the control sample in CD3^-^, CD56^-^ B cells (**Figure 6E**). Monocytes in the PBL were gated based on the FSC-H and SSC-H, and also showed no significant migration toward any of the moLC supernatants (**Figure 6F**), and indeed tended to migrate more toward the vehicle control (supplemented RPMI, indicated by the dashed line across all graphs; **Figures 6B–F**).

## 3 Discussion

In this study, we aimed to investigate the effects of BNP on the function of moLCs under various TLR stimuli. Based on our previous findings, we showed that BNP present during differentiation modulated the moLCs gene set toward a migratory subtype (32). In the present work, we extended these observations by incorporating a 24-hour activation step to examine the LCs’ function in the inflammatory response. We chose two endosomal TLR ligands, a TLR7/8 agonist CL075 and a TLR3 agonist poly(I:C).

CL075 is a resiquimod analog that triggers NF-κB and IRF pathways through TLR7/8, which typically results in pro-inflammatory cytokine production (35). CL075 has the most significant effect on the main characteristic marker of LCs, langerin/CD207 (**Figures 1A, B**). This C-type lectin shares similar carbohydrate ligands with DC-SIGN, and also binds HIV-1, contributing to virus degradation with the induction of autophagy to protect LCs from infection. Thus, its induction by a TLR7/8 ligand most likely reflects an antiviral response (36). Furthermore, CL075 activation significantly increased HLA-DQ expression (**Figure 1C**), which was also observed in the case of plasmacytoid dendritic cells (pDCs) where it also increased expression of CD80, CD86 and CD40 (37).

Poly(I:C) is a double-stranded RNA analog that can be sensed by the TLR3 receptor. The presence of TLR3 in the skin serves not only to sense viruses, but also to promote genes that support barrier-repair mechanisms in keratinocytes after UV injury (38). In addition to TLR3, poly(I:C) can bind to retinoic acid-inducible gene I (RIG-I) and melanoma differentiation-associated protein 5 (MDA5) (39, 40). In our experimental system, poly(I:C) did not significantly affect the LCs’ main markers, i.e. CD1a and CD207 (**Figures 1A, B**). When we examined the activation markers CD86 and HLA-DQ, poly(I:C) treatment significantly increased the positive cells (**Figures 1C, D**), which correlates with the response of epidermal LCs to poly(I:C) treatment, where it also had a similar impact on costimulatory markers (41). In another study, the clinically applied form stabilized poly(I:C) (s-poly(I:C)) was used on PBMC-derived LCs, where they experienced the same effect on costimulatory markers and MHC molecules (42). Increased costimulatory molecules on antigen-presenting cells (APCs) are connected to the induction of T cell activation and proliferation. The T cell proliferation−inducing effects of poly(I:C) were supported in MLR using s-poly(I:C) on HPV-exposed LCs (42). In contrast, under our experimental conditions, poly(I:C) did not significantly impact T cell proliferation induction (**Figure 3A**).

Combined stimulation with CL075 and poly(I:C) produced the most striking effect on moLC activation markers, cytokine production, T cell proliferation, and lymphocytes migration (43). Upon a combined TLR activation, production of proinflammatory cytokines IL-6, IL-8, and TNF-α was significantly increased compared to using CL075 or poly(I:C) alone (**Figure 2**). This effect of TLR agonists was significantly decreased when the cells were treated with BNP during their differentiation, hinting at a potential anti-inflammatory effect of BNP. Notably, the production of anti-inflammatory IL-10 was also diminished by the presence of BNP (**Figure 2D**). IL-10 is a key regulator that restrains excessive inflammation by limiting dendritic cell and macrophage activation and by dampening T cell effector responses, thus contributing to peripheral tolerance and protection from tissue damage. At the same time, IL-10 can also promote immune suppression by reducing antigen presentation and T cell proliferation, which may impair effective pathogen clearance in some settings (44–46). In our system, BNP-treated moLCs displayed a broad reduction in proinflammatory cytokines and T cell stimulatory capacity, suggesting that BNP primarily exerts an overall inhibitory effect on moLC activation despite lowering IL-10. We therefore speculate that, in this context, reduced IL-10 production may slightly lessen IL-10–mediated immune suppression without fully abolishing BNP-driven restraint of inflammatory cytokine secretion, underscoring the nuanced balance between immune regulation and immune suppression in BNP-conditioned moLCs. In contrast, an *in vitro* study with RAW 264.7 macrophages showed that BNP reduced IL-6 and TNF-α production and increased IL-10 secretion by suppressing NF-κB and MAPK pathways (47). In these experiments BNP was applied after LPS activation, rather than during the differentiation of the cells, which is the most likely explanation to the differing effects on similar endpoints. The impact of the BNP receptor on pro-inflammatory cytokine production was also examined in NPR1 knock-out mice, where it was reported to increase IL-6, TNF-α, and IL-1β in the heart ventricle (48). Despite these pro-inflammatory effects of BNP, it has also been shown to increase IL-10 release in human THP-1 macrophages (49). In conventional type 1 dendritic cells (cDC1s) and conventional type 2 dendritic cells (cDC2 cells), BNP treatment reduces caspase-1 activation and IL-1β production through the NPR1 receptor (12). Taken together, these findings underscore the highly context-dependent immunomodulatory effects of BNP, prompting us to define how these TLR agonists influence moLCs’ cytokine production in the context of viral infection. The mixture of TLR 3 and TLR 7/8 agonists has already been used to activate mature DCs (mDCs) and moLCs, both of which can be induced to secrete high levels of IL-12 (43, 50). As expected poly(I:C) and CL075 promoted IL-12 and IFN-β secretion, which were reduced by BNP. These results demonstrate that BNP cannot be considered either pro- or anti-inflammatory, rather it seems to generally inhibit cytokine secretion.

To determine what the net effect of BNP on moLCs antigen presenting function is, we next investigated the T cell activation capacity of the cells (**Figure 4**). Poly(I:C) and CL075 have been described to increase the ability of moLCs and monocyte-derived dendritic cells (moDCs) to induce T cell proliferation and to polarize Th1 cells (43, 50). We found that moLCs differentiated in the presence of BNP showed significantly decreased ability to induce proliferation after their activation with TLR agonists. A likely cause for this effect was the increase in IDO mRNA expression shown in our previous data (32), however poly(I:C) and CL075 caused comparable increases on the protein level, indicating that IDO1 alone is insufficient to explain the BNP-mediated reduction in T cell proliferation. Although we examined BNP effects on moLCs cytokine production and T cell proliferation induction ability we cannot infer its impact on T cell polarization in the current data.

Our data suggests that BNP does not merely act as a general immunosuppressant but rather orchestrates a specific trade-off between migration and inflammation. Typically, DC activation links migration (CCR7) with high cytokine secretion to prime potent T cell responses. However, BNP treatment uncoupled these processes: it enhanced chemotaxis toward CCL19/21 but potently suppressed the secretion of Th1-polarizing cytokines (IL-12) and neutrophil-recruiting chemokines (CXCL8). This profile resembles a ‘resolution’ phenotype, where the priority shifts from promoting inflammation to clearing immune cells from the tissue. While this interpretation aligns with the observed gene expression and functional patterns, additional mechanistic studies will be required to determine whether BNP directly programs this phenotype or indirectly influences it through broader regulatory pathways. To gain deeper insight into the effect of BNP on moLCs in the context of their activation with TLR ligands we performed RNA-Seq experiments where we compared TLR-ligand activated cells with moLCs differentiated in the presence of BNP. According to our data (**Figure 4**.) the two groups upregulated similar genes, but the latter cells showed a lower degree of increase in expression of pro-inflammatory genes, while also downregulating some important cytokines and chemokines. The previously described shift toward a migratory phenotype was also apparent after the activation of the cells, which prompted us to more closely investigate the migratory capacity of these cells, as well as their ability to influence the migration of other immune cells. BNP binding to their receptor NPR1, generates increased intracellular cGMP levels (51), which we confirmed in our moLC model (32). Phosphodiesterase 5 (PDE5), originating from a progressed tumor, degrades cGMP, which negatively regulates the motility of the cells. This suggests that cGMP promotes DC migration via cytoskeletal rearrangements, without affecting CCR7 expression (52).

Activated LCs migrate from the skin to secondary lymphoid organs, where they subsequently activate naïve T cells, inducing their proliferation. Lymphatic endothelial cells produce CCL19 and CCL21 to drive LCs into the lymphatic vessels (28). In our results, CCR7 agonists increased the migration of BNP treated moLCs, especially toward CCL21, which correlates with BNP promoting a migrating phenotype of LCs (32).

Using supernatant from TLR-activated moLCs increased the migration of PBL, especially CD3^-^CD56^+^ NK cell population (**Figures 6B, D**). Combined TLR agonist application in moDCs increased IL-12 production and NK cell activation capacity, which was reflected in NK cells’ IFNγ production and cytotoxic activity (50). This migration was significantly inhibited by supernatant from BNP-pretreated and activated moLCs, which is potentially related to the influence of BNP on moLCs IL-12 production (**Figures 2E**, **6D**). The RNA-Seq and chemokine array findings support this functional outcome. TLR activation resulted in an elevation of several chemokines, such as CCL1, IL-8, CCL5, CXCL10, CXCL11, CCL20, CXCL1, and CCL19 (**Figures 5B, C**).

CCL1 is a chemokine ligand of the CCR8 receptor, which facilitates a selective homing mechanism for the infiltration of αβ T cells into the skin during steady state (53, 54). There are CCR8^+^ NK cells in the skin that migrated toward CCL1 in Transwell migration assay, in contrast to NK cells from peripheral blood, which lack CCR8 (54). Although BNP reduces CCL1 expression (**Figures 5B, C**), this does not explain the reduced migration of NK cells from peripheral blood (**Figure 6D**). In psoriatic skin, NK cells highly express CXCR3 and CCR5, and their ligands CXCL10 and CCL5 drive migration of NK cells and may contribute to the worsening of the disease (55). CD56^+high^ NK cells from peripheral blood express high levels of CXCR3 and CCR5. Poly(I:C) and CL075 facilitate the production of chemokine receptor ligands, and they may influence NK cell migration in our experiments. In contrast, BNP treatment inhibited the production of CXCL9, CXCL10, and CXCL11 (**Figures 4C**, **5B, C**). These chemokines are important immune chemoattractants in interferon induced immunological responses, as is their receptor CXCR3, which is also substantially expressed on activated T cells (56). BNP decreased CCL20 production in moLCs. CCL20 is a chemoattractant for T cells and dendritic cells, and may contribute to the recruitment of T cells in psoriatic skin (57).

While the link between BNP and AD is more firmly established as detailed above, the potential relevance of the BNP pathway to the pathophysiology of psoriasis warrants careful consideration. Psoriasis is inherently linked to an increased risk of cardiovascular comorbidities; consequently, N-terminal pro-B-type natriuretic peptide (NT-proBNP) has emerged as a critical biomarker for monitoring this risk and screening for subclinical cardiac dysfunction within this patient population (58–60). Elevated systemic levels of NT-proBNP are frequently observed in patients with severe psoriasis, psoriatic arthritis, and established cardiovascular disease. However, it remains to be elucidated whether this elevation is purely a systemic manifestation of cardiac strain or if BNP is expressed locally within psoriatic skin lesions. If present in the skin, BNP may contribute directly to plaque formation and local inflammation, mirroring the mechanisms recently described in atopic dermatitis.

In conclusion, BNP appears to influence moLC function by promoting their migratory activity toward lymph nodes and modulating their inflammatory profile. We hypothesize that BNP supports this role by reducing cytokine and chemokine secretion, T cell proliferation induction ability, and NK-cell stimulatory capacity during inflammatory or viral stimuli.

One limitation of our study is that we used moLCs instead of *in vivo* tissue LCs. Although moLCs share many phenotypic features with resident cells, their gene expression profile and maturation dynamics may differ from those naturally found in the skin, which may affect the interpretation of the immune response. Donor−to−donor variability can introduce substantial variation between samples, which makes statistical analyses more challenging. Although cytokine production can be used to infer potential effects on T cell polarization, we did not directly assess T cell subset differentiation in this study, and further experiments are needed. The presence of BNP in the skin has been described, but its role in modulating cutaneous immune cells *in situ* remains unexplored.

## 4 Materials and methods

### 4.1 Isolation of monocytes and differentiation of moLCs

Buffy coats enhanced with heparinized leukocytes were obtained from healthy volunteers. Both the Head of the National Transfusion Service and the Regional and Institutional Ethics Committee of the University of Debrecen’s Faculty of Medicine (Debrecen, Hungary) approved the procedure, as well as the Regional Blood Center of the Hungarian National Blood Transfusion Service (Debrecen, Hungary; approval number: OVSZK 3572-2/2015/5200). Gradient centrifugation was used to collect PBMCs from human blood samples using Ficoll-Paque™ Plus (Cytiva, Marlborough, MA, USA). Monocytes were isolated from PBMCs using anti-human CD14^+^ microbead (Miltenyi Biotech, Bergisch Gladbach, Germany). RPMI 1640 medium (Thermo Fisher Scientific, Waltham, MA, USA) containing 10% heat-inactivated FBS, 10 mM HEPES, 50 mM 2-Mercaptoethanol, 1% penicillin (all from Sigma-Aldrich, St. Louis, MI, USA) were used for the culture of CD14^+^ monocytes. The cells were plated in 12-well tissue-culture plates (Techno Plastic Products, Trasadingen, Switzerland) at 1x10^6^ cells/ml. Media was supplemented with GM-CSF 200 ng/mL (Gentaur Molecular Products, London, UK) TGF-β (10 ng/mL), TNF-α (20 ng/mL), and IL-4 (20 ng/mL for the first 48 hours) for 5 days at 37 °C (the other cytokines from PeproTech, Rocky Hill, NJ, USA) to induce moLCs differentiation. MoLCs were treated with 10 nM BNP on day 0, day 2, and day 4. MoLCs were activated with 0.5 µg/ml CL075 (TLR7/8 agonist) and 20 µg/ml poly(I:C) (TLR3 agonist) and with their combination at day 4 for 24 hours (both were obtained from Invivogen, San Diego, CA, USA).

### 4.2 Flow cytometry

MoLCs were collected on day 5, washed with FACS buffer containing PBS 2 (v/v) % heat-inactivated FBS and 2 mM EDTA (pH 7.4) and stained with the following antibodies: CD1a-Phycoerythrin (PE), CD207-Allophycocyanin (APC), CD83-Fluorescein Isothiocyanate (FITC), (all from BioLegend, San Diego, CA, USA), CD86-PE (R&D Systems, Wiesbaden, Germany), and HLA-DQ-APC (Thermo Fisher Scientific) for 20 minutes on ice.

Stained cells were measured with an ACEA NovoCyte 2000R cytometer (ACEA Biosciences, San Diego, CA, USA) and results were analyzed with FlowJo 10.8.1 software (BD Biosciences, Franklin Lakes, NJ, USA).

### 4.3 Enzyme-linked immunosorbent assay

Supernatants from cells were collected at day 5 by centrifugation. Cytokine concentrations were determined with ELISA kits for IL-6, IL-8, IL-10, and TNF-α by BD Biosciences and IL-12, IFN-β by R&D Systems, following the manufacturer’s protocol. The EnVision 2105 Multimode Plate Reader (PerkinElmer, Waltham, MA, USA) was used to analyze cytokine levels, and concentrations were calculated using GraphPad Prism 9.1.2 for Windows (GraphPad Software) using a cubic logistic model.

### 4.4 T cell proliferation

Naïve CD4^+^ T cells were isolated from human buffy coat using the Naïve CD4^+^ Isolation Kit (Miltenyi Biotec), following the manufacturer’s protocol. CD4^+^ T cells were stained with 0.5 μM carboxyfluorescein succinimidyl ester dye (CFSE) (Thermo Fisher Scientific) and were cocultured with moLCs in 1:10 ratio using round bottom 96 well plate. The coculture RPMI 1640 medium was supplemented with 1 μg/ml anti-human CD3 mAb to activate the T cells (BD-Biosciences, San Jose, CA, USA). Cells in the coculture were collected at day 5 and CFSE intensity was measured by flow cytometry using an ACEA NovoCyte 2000R cytometer (ACEA Biosciences).

### 4.5 Western blot

Cells were collected in a detergent mix to lyse cells, and proteins (30 µg) were loaded to SDS-PAGE (10% gels) and transferred to nitrocellulose membranes (Bio-Rad, Hercules, CA, USA). Membranes were blocked with 5% non-fat milk powder solution and incubated with primary antibody against IDO1 (Thermo Fisher) overnight at 4 °C. A horseradish peroxidase-conjugated goat anti-mouse IgG (Thermo Fisher) was used as a secondary antibody to detect the primary antibody (Thermo Fisher Scientific), and protein bands were visualized with SuperSignal West Pico Chemiluminescent Substrate-enhanced chemiluminescence kit (Thermo Fisher Scientific) using an Azure c300 imaging system (Azure Biosystems, Dublin, CA, USA). Anti-human beta-actin mAb (Bio-Rad) was used as a loading control.

### 4.6 RNA-seq

MoLC samples were collected on day 5 for high throughput mRNA Illumina sequencing analysis. RNA integrity was measured with Agilent BioAnalyzer using an Eukaryotic Total RNA Nano Kit (Agilent Technologies, Waldbronn, Germany). The applicable RNA integrity number was 7 for the library preparation. Ultra II RNA Sample Prep kit (New England BioLabs Inc., Ipswich, MA, USA) was used for the libraries. An Illumina NextSeq 500 instrument (Illumina, Inc., San Diego, CA, USA) was used for sequencing. Statistical analysis was performed with StrandNGS software (https://www.strand-ngs.com). Library preparation, sequencing and primary data analysis were carried out at the Genomic Medicine and Bioinformatics Services Laboratory of the University of Debrecen. Further evaluation was made using the Galaxy web platform (usegalaxy.org). Volcano plots were generated with the help of the VolcaNoseR web app (61). Gene set enrichment analysis was performed using the ShinyGO web app (61).

### 4.7 Chemokine array

Supernatants were collected from moLCs at day 5. Proteome Profiler Human Chemokine Array Kit (R&D Systems) was performed according to the manufacturer’s instructions. The Azure c300 imaging system (Azure Biosystems) was used to analyze chemiluminescence signals from the membranes. Densitometry was performed using Fiji (62).

### 4.8 Chemotaxis assay

Cell migration was analyzed with 24-well Transwell plate (Corning, New York, NY, USA). The lower wells were filled with RPMI medium supplemented with CCL19 and CCL21 (200 ng/ml). RPMI without chemoattractant was used as a control for measuring random chemotaxis. MoLCs suspension (1 x 10^6^ cells/ml) was taken to the upper wells. Cells were incubated in Transwell for 1 hour at 37 °C. After incubation, the cells from the lower wells were scraped and collected. After centrifugation, FACS buffer was added to the cells. Migrating cell numbers were measured by flow cytometry in a fixed volume of 50 µl.

### 4.9 PBL migration

PBL migration was analyzed with 24 well Transwell plates (Corning). PBL were isolated from human buffy coat with negative separation using CD14^+^ microbeads. Cells were centrifuged in 500 x g for 5 minutes and incubated for 1 hour in RPMI 1640 medium (Thermo Fisher Scientific) containing 10% heat-inactivated FBS, 10% HEPES, 50 mM 2-Mercaptoethanol, 1% penicillin (all from Sigma-Aldrich) in T75 flasks to remove residual monocytes from the cell suspension. MoLCs supernatant (CTRL, BNP, Poly(I:C)+CL075, BNP+Poly(I:C)+CL075) was utilized in the lower wells of the Transwell plate. PBL suspension in RPMI 1640 medium was taken to the upper wells using 1x10^6^/well cell count. Cells were incubated in Transwell for 1 hour at 37 °C. The cells from the lower wells were scraped and collected after incubation. FACS buffer was applied to the cells following centrifugation. Cells were stained with CD3-FITC (BioLegend) and CD56-PE (Immunotech, Marseille, France). Migrating cells were determined by flow cytometry in a fixed volume of 50 µl.

### 4.10 Statistical analysis

Individual statistical analyses were performed using GraphPad Prism 9.1.2. for Windows (GraphPad Software, San Diego, CA, USA). Two-sample comparison of 2 groups, two-sided, unpaired Student’s t-test, comparing three or more groups analyzed by One-way ANOVA followed by or Two-way ANOVA followed by Tukey’s multiple comparison test applied, depending on the experimental design. The assumptions for parametric analysis were rigorously assessed: for One-way ANOVA, the homogeneity of variances was verified using the Brown-Forsythe test, while for Two-way ANOVA, it was confirmed via diagnostic analysis of the residuals. Based on the QQ plots, the distribution of the residuals followed a normal distribution and the model fit was adequate. Differences were considered statistically significant at P < 0.05. The differences were considered statistically significant for P < 0.05.

## Data Availability

The datasets presented in this study can be found in online repositories. The names of the repository/repositories and accession number(s) can be found below: https://www.ncbi.nlm.nih.gov/geo/, GSE291916.
