# Supplementary material for: B-type natriuretic peptide attenuates TLR-induced cytokine and chemokine secretion in monocyte-derived Langerhans cells
Source: Front Immunol. 2026 Mar 19;17:1791284. doi: 10.3389/fimmu.2026.1791284 (PMC13043391; doi:10.3389/fimmu.2026.1791284)
Supplement: Supplementary file 1 [file DataSheet1.pdf]

Supplementary Material

1.1 Supplementary Figures

Supplementary 1

A

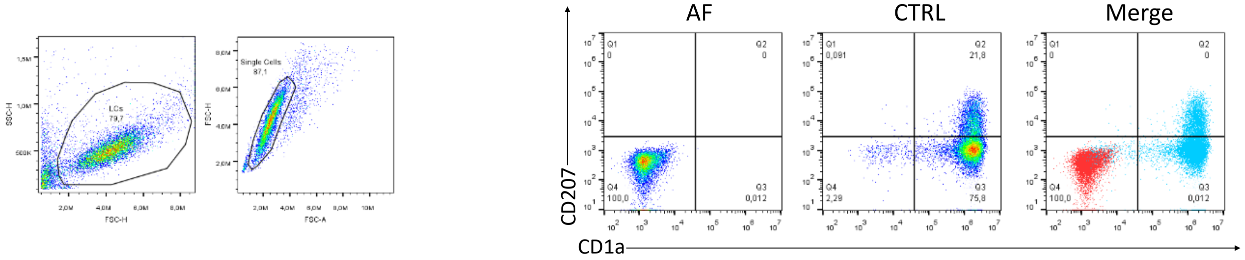

B

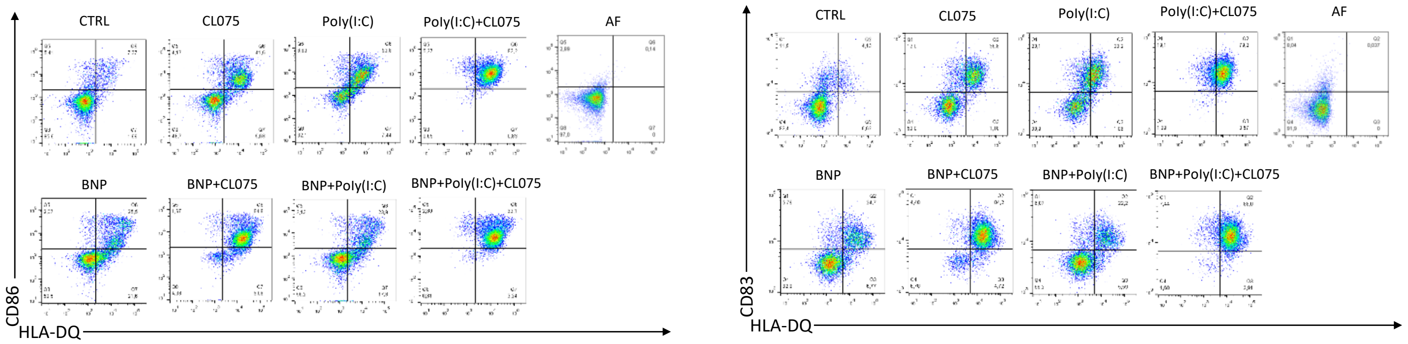

Supplementary Figure 1:

(A) Flow cytometry gating strategy was used to define the moLCs population. MoLCs were gated based on FSC-H and SSC-H, and single cells were selected from FSC-A versus FSC-H dot plot. The quadrant in the density plot of CD1a and CD207 markers was adjusted to autofluorescent cells. The same gating strategy was applied to CD86, CD83, and HLA-DQ markers. **(B)** Representative density plot of the costimulation and activation markers of moLC CD83, CD86 and HLA-DQ expression measured by flow cytometry. Monocytes were cultured in the presence of GM-CSF, TNF-α, and TGF-β for 5 days supplemented with IL-4 for the first 48 hrs to differentiate moLCs, and treated with 10 nM BNP throughout the entire differentiation process. On day 4 moLCs were activated with CL075 and poly(I:C) and a combination of both for 24 hrs. BNP, B-type natriuretic peptide; CL075, TLR7/8 agonist, thiazoquinoline compound; CTRL, Control; Poly(I:C), TLR3 agonist, polyinosinic:polycytidylic acid

Supplementary 2

A

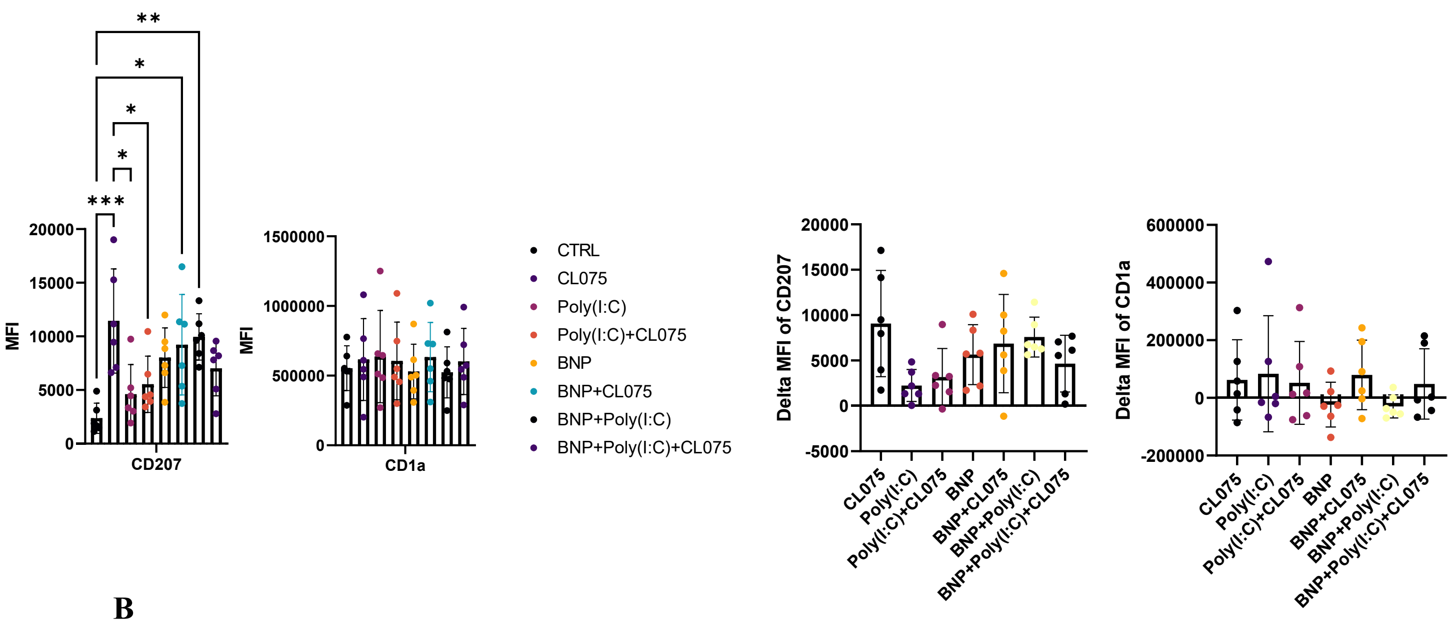

B

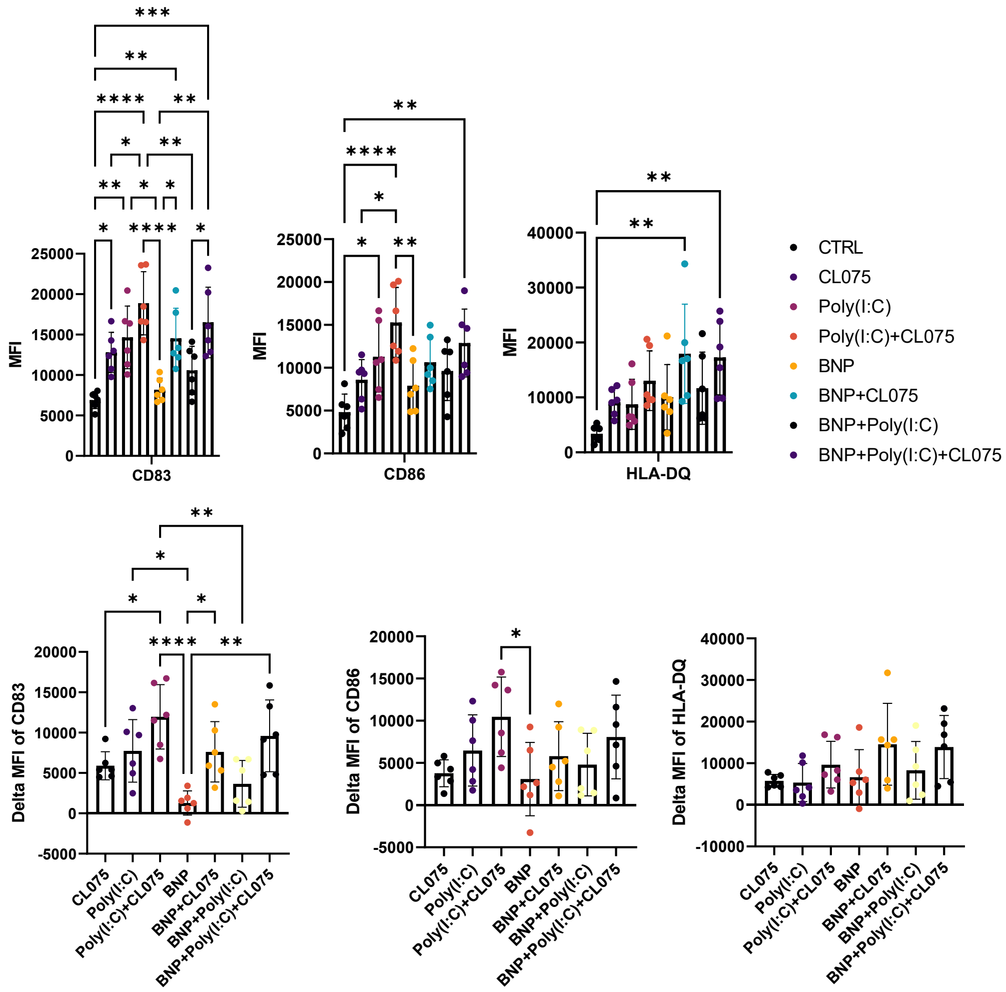

Supplementary Figure 2:

MFI and delta MFI of the main markers of moLCs **(A)** CD1a and CD207 **(B)** costimulation and activation markers CD83, CD86 and HLA-DQ. Monocytes were cultured in the presence of GM-CSF, TNF-α, and TGF-β for 5 days supplemented with IL-4 for the first 48 hrs to differentiate moLCs, and treated with 10 nM BNP throughout the entire differentiation process. On day 4 moLCs were activated with CL075 and poly(I:C) and a combination of both for 24 hrs. BNP, B-type natriuretic peptide; CL075, TLR7/8 agonist, thiazoquinoline compound; CTRL, Control; MFI, Mean fluorescent intensity; Poly(I:C), TLR3 agonist, polyinosinic:polycytidylic acid; *P<0.05, **P<0.01, **** P< 0.0001.

Supplementary 3

A

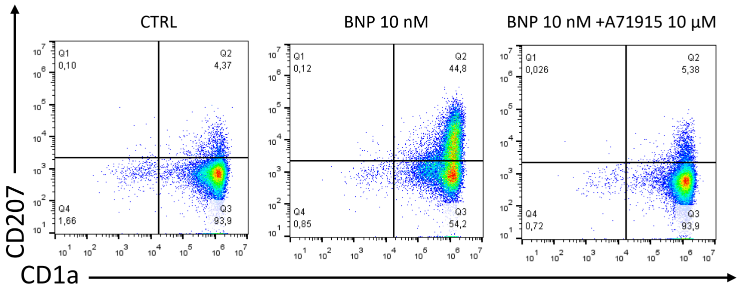

B

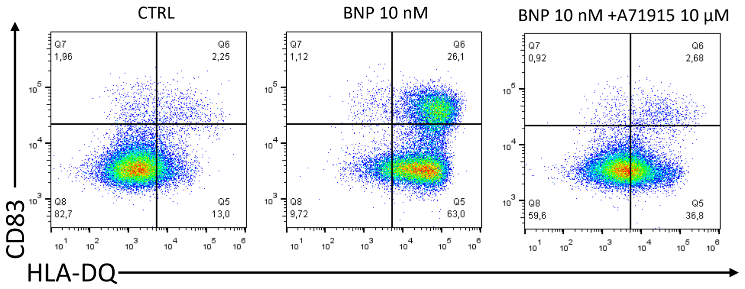

C

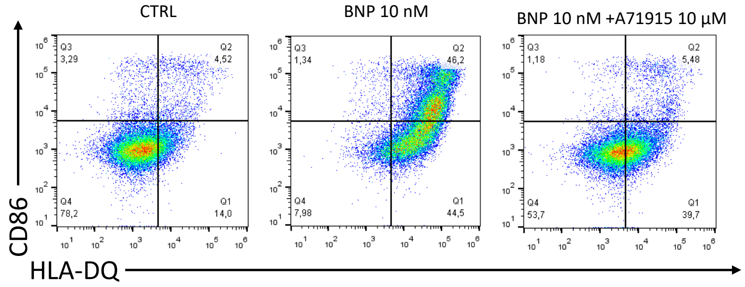

Supplementary Figure 3:

Representative density plot of differentiation markers of moLC **(A)** CD207 and CD1a, and costimulation and activation markers **(B-C)** CD83, CD86 and HLA-DQ expression measured by flow cytometry. Monocytes were cultured in the presence of GMCSF, TNF-α, and TGF-β for 5 days supplemented with IL-4 for the first 48 hrs to differentiate moLCs, and treated with 10 nM BNP and 10 µM A71915 throughout the entire differentiation process. On day 4 moLCs were activated with CL075 and Poly(I:C) and a combination of both for 24 hrs. A71915, competitive NPRA antagonist; BNP, B-type natriuretic peptide; CL075, TLR7/8 agonist, thiazoquinoline compound; CTRL, Control; Poly(I:C), TLR3 agonist, polyinosinic:polycytidylic acid;
